# Individual and additive effects of childhood maltreatment and substance use disorder histories on baseline and stress-induced changes in peripheral stress biomarkers

**DOI:** 10.1007/s00213-025-06953-1

**Published:** 2025-12-01

**Authors:** Abigail R. Lunge, Lars Östman, Ryann Tansey, Daniel J. O. Roche, Elisabeth R. Paul, Andrea J. Capusan, Markus Heilig, Leah M. Mayo

**Affiliations:** 1 Mathison Centre for Mental Health Research and Education, Hotchkiss Brain Institute, Department of Psychiatry, University of Calgary, Calgary, Canada; 2 Center for Social and Affective Neuroscience, Linköping University, Linköping, Sweden; 3 Maryland Psychiatric Research Center, Department of Psychiatry, University of Maryland Baltimore, Baltimore, MD USA

**Keywords:** Kynurenine pathway, Endocannabinoid, Childhood maltreatment, Substance use disorder, Stress

## Abstract

**Background:**

Exposure to childhood maltreatment (CM) has serious consequences on the health of affected individuals, potentially elevating vulnerability to various psychopathologies, including substance use disorders (SUDs). Recent investigations have implicated several biological signaling systems in vulnerability to SUD development following CM, including the kynurenine (KYN) pathway and endocannabinoid (eCB) system. Potential crosstalk between these systems has scarcely been explored.

**Methods:**

The present exploratory analysis investigated the relationship between baseline and stress-induced changes in eCBs, KYN metabolites, inflammatory biomarkers, and cortisol across CM and SUD status (CM + SUD, CM only, SUD only, and healthy controls) using a factor analysis. Participants (*N* = 101) completed an acute laboratory stressor and blood samples were collected at five-timepoints throughout the task.

**Results:**

Factor analysis revealed that KYN metabolites explained the majority of total variance in the dataset. The pro-inflammatory marker CRP was associated with neurotoxic KYN metabolites. Subsequent group-level analyses revealed that CM status significantly impacted a pro-inflammatory factor (baseline and stress-induced changes in CRP and IL-6). Additionally, CM and SUD status exhibited an interaction effect on a factor primarily comprised of 2-AG at baseline and throughout stress, such that in absence of CM, SUD was associated with significantly reduced levels of 2-AG.

**Conclusions:**

Exposure to CM is associated with pro-inflammatory states at baseline and across stress exposure. Additionally, 2-AG may be a marker of SUD pathology in the absence of CM. However, no effect of CM or SUD status was found on KYN pathway metabolites. The mechanisms underlying elevated susceptibility to SUD following CM-exposure require further investigation.

**Supplementary Information:**

The online version contains supplementary material available at 10.1007/s00213-025-06953-1.

## Introduction

Childhood maltreatment (CM), defined as physical, sexual, or emotional abuse, and/or neglect, has long-term consequences on the mental and physical health of affected individuals, including increased risk of stress-related psychopathologies like substance use disorder (SUD) (Capusan et al. 2021; Enoch 2011; Hyman et al. 2008; Schwandt et al. 2013). Notably, CM is associated with an earlier age of SUD onset and greater disorder severity (Anda et al. 2006; Dube et al. 2006; Guastaferro and Shipe 2023; Huang et al. 2012; Schwandt et al. 2013; Stocchero et al. 2024). The poorly understood influence of early life experiences on the development of SUDs and the resulting diverse patient population poses a challenge for SUD treatments. Understanding the underlying correlates of how early life experiences, such as CM, contribute to the development of SUD is crucial for the elucidation of therapeutic targets and interventions.

Recent work has explored mechanisms that may confer susceptibility or resilience to SUD development following CM exposure, including the endocannabinoid (eCB) system. The eCB system is a neuromodulatory system integral to various brain functions, including the stress response (Balsevich et al. 2017; Mayo et al. 2020; Morena et al. 2016), emotional processing (Paulus et al. 2021; Petrie et al. 2021), and inflammation (Almogi-Hazan and Or 2020). During instances of acute stress, eCB signaling modulates appropriate physiological responses, such as activation of the hypothalamus-pituitary-adrenal (HPA) axis and immune responses (Almogi-Hazan and Or 2020; Micale and Drago 2018). Exposure to chronic and traumatic stressors such as CM, however, has significant consequences on these processes (for comprehensive review see Danese and McEwen 2012). For instance, children and adults exposed to CM exhibit elevated basal levels of CORT, indicative of HPA axis dysfunction (Cicchetti and Rogosch 2001). Moreover, individuals with history of CM display blunted neuroendocrine responses to psychosocial stress tasks (MacMillan et al. 2009). Additionally, eCB signaling plays a modulatory role in inflammatory responses to stress and may mediate enhanced pro-inflammatory signaling observed in CM populations (Almogi-Hazan and Or 2020; Danese and McEwen 2012). Dysregulation of eCB-modulation of stress and immune responses may thereby confer vulnerability to developing stress-related psychopathologies like SUD and serve as a novel therapeutic target.

Recent findings from our group support this, demonstrating that eCB signaling differs between individuals with a prospectively documented history of CM without a current or lifetime SUD diagnosis (operationally defined as “resilient”) and those with a history of both CM and SUD diagnosis, implicating eCB signaling as a potential mediator of resilience to SUD pathology in these populations (Perini et al. 2023). The precise biological mechanisms through which eCB signaling moderates the risk of SUD development, however, remain poorly understood.

**Fig. 1:**
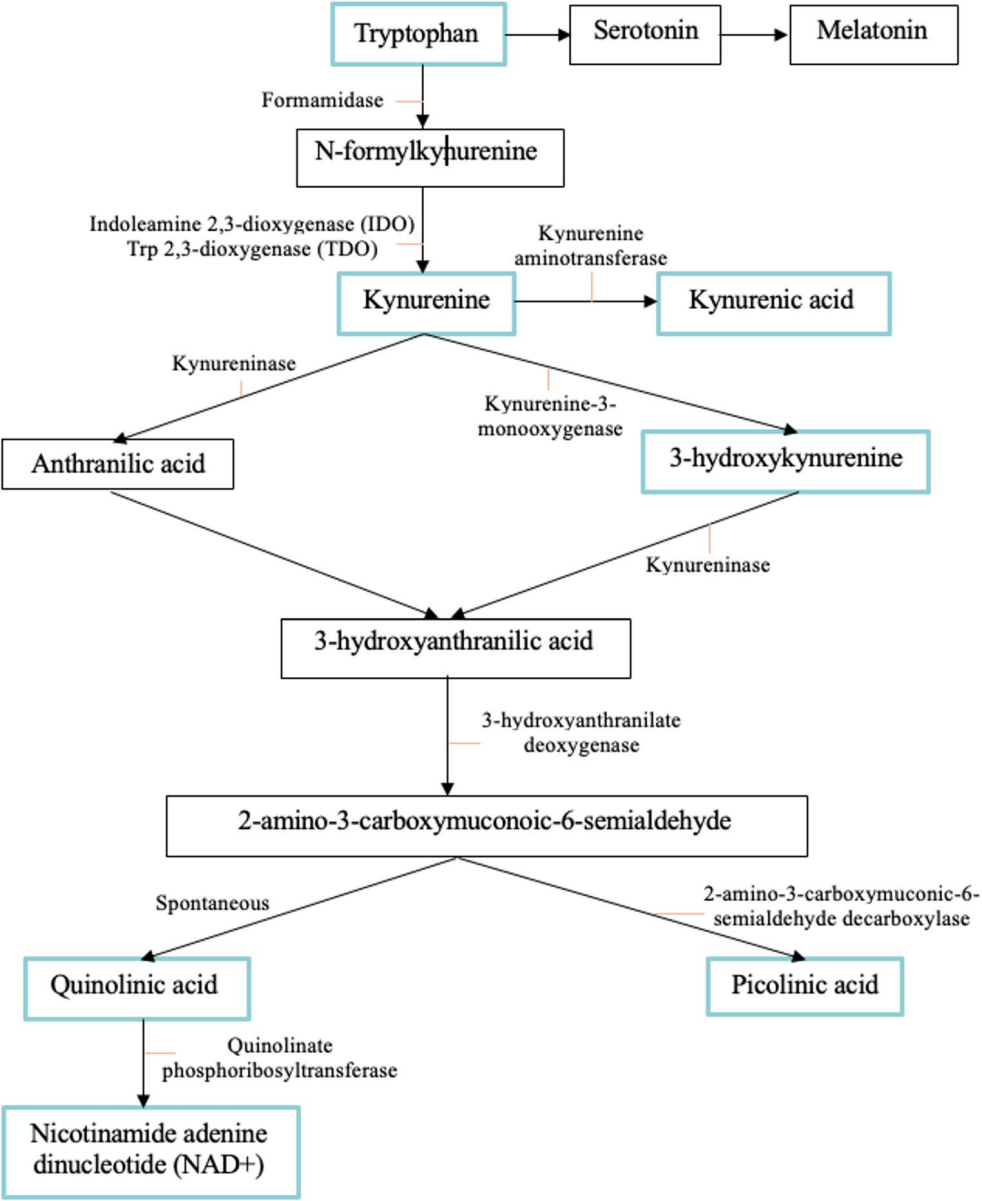
The kynurenine pathway. The metabolic pathways by which tryptophan (Trp) is degraded into either serotonin or kynurenine (KYN). Metabolites measured in the present study are boxed in blue

The kynurenine (KYN) pathway (Fig. 1), the primary metabolic pathway through which tryptophan (Trp) is degraded, has likewise received interest as a novel mechanism and therapeutic target for SUD pathology (Giménez-Gómez et al. 2018; Justinova et al. 2013; Secci et al. 2017; Vengeliene et al. 2016). KYN is metabolized through one of two branches along the KYN pathway: along one, often termed the “anti-inflammatory” or “neuroprotective” path, KYN is metabolized into kynurenic acid (KYNA). Along the other, generally referred to as the “inflammatory” or “neurotoxic” pathway, KYN metabolism cumulates in quinolinic acid (QUIN), an NMDA receptor agonist (Stone and Perkins 1981), that can induce over-excitation of the NMDA receptor and promote neuronal influx of Ca^2+^, ultimately triggering the activation of apoptotic pathways and cell death (Choi 1987; Hestad et al. 2022; Schwarcz et al. 1983; Zhou and Sheng 2013). Elevations in QUIN have been implicated in various mood and degenerative disorders (for comprehensive review, see Hestad et al. 2022; Schwarcz et al. 2012). Conversely, KYNA, an NMDA receptor antagonist and α7- nicotinic acetylcholine receptor negative allosteric modulator, has neuroprotective and anticonvulsant properties (Foster et al. 1984; Perkins and Stone 1982; Russi et al. 1992), preventing neuronal loss following excitotoxic, ischemia-induced, and infectious neuronal injuries (Vamos et al. 2009). Thus, shifts in KYN pathway metabolism in favour of the neurotoxic branch may thereby contribute to psychopathology development and represent a novel therapeutic target.

Furthermore, as with the eCB system, neuroendocrine and immune signaling are closely related to KYN pathway metabolism. The degradation of Trp into KYN involves the enzymes indoleamine 2,3-dioxygenase (IDO) and Trp 2,3-dioxygenase (TDO); IDO is upregulated by pro-inflammatory signaling molecules stemming from an innate immune response, while TDO activity is induced by Trp itself, corticosteroids, glucagon, and immune activation (Comai et al. 2020; Lashgari et al. 2023; O’Connor et al. 2009; Salter and Pogson 1985). Thus, history of CM or SUD diagnosis, which is associated with enhanced inflammatory signaling (Agarwal et al. 2022; Baumeister et al. 2016; Coelho et al. 2014) and dysregulated glucocorticoid responses to stress, (Lijffijt et al. 2014; Marques-Feixa et al. 2023; Zhong et al. 2019) may activate these enzymes, altering KYN pathway metabolism.

Indeed, existing research has demonstrated an association between KYN pathway metabolism, chronic stress exposure, and stress-related psychopathologies such as SUD (Comai et al. 2024; Contella et al. 2024; Leclercq et al. 2021; Wences Chirino et al. 2023). A recent meta-analysis investigating the KYN pathway in alcohol use disorder reported increased peripheral levels of KYN and reductions in KYNA in individuals with alcohol use disorder compared to healthy controls (Wang et al. 2023). Similarly, elevations in QUIN and reductions in KYNA have been observed in patients with alcohol use disorder relative to healthy controls (Leclercq et al. 2021). Taken together, this evidence poses the KYN metabolic pathway as an interesting target to investigate in CM and SUD populations.

Furthermore, crosstalk between the eCB system and KYN pathway through neuroendocrine and immune signaling may serve as an important mechanism contributing to enhanced vulnerability to SUD in CM populations. Preclinical work has provided supporting evidence for interaction between the KYN pathway and eCB system, with KYN pathway metabolism modulating the eCB-mediated effects of delta 9-tetrahydrocannabinol, the psychoactive component of cannabis (Beggiato et al. 2022; Bilel et al. 2025; Justinova et al. 2013). To date, minimal investigation of the impact of CM exposure and SUD pathology status on these systems and their relationship has been conducted.

The present secondary analysis addressed this by assessing the impact of CM and SUD status on peripheral measures of KYN pathway metabolites, eCBs, inflammatory biomarkers, and cortisol. We assessed peripheral levels of these molecules at baseline and in response to an acute stressor, expanding upon our previously reported findings that the eCB system, specifically elevated levels of peripheral AEA at baseline and throughout stress exposure, may confer resilience to SUD pathology in CM populations (Perini et al. 2023). The aim was to see how these biomarkers interact in the context of CM and/or SUD status, potentially identifying novel biomarkers or therapeutic targets.

We hypothesized that prospectively documented exposure to CM would be associated with higher levels of KYN metabolites generated along the “inflammatory branch” of the KYN pathways, elevated pro-inflammatory signaling, and dysregulated eCB signaling at baseline and in response to an acute laboratory stressor. Likewise, we hypothesized that a lifetime or current SUD diagnosis would be correlated with greater KYN inflammatory branch metabolites and enhanced pro-inflammatory signaling at baseline and throughout stress exposure, as well as a blunted eCB response to an acute stressor. Finally, we hypothesized that the interaction of CM and SUD would have additive effects on these outcomes.

Of note, the present study utilized prospective documentation of CM (e.g., physical, sexual abuse, and/or severe neglect) in children or adolescents who were referred to a specialized Trauma Unit within the Child & Adolescent Psychiatry Department of Linköping University Hospital. All participants additionally completed the Childhood Trauma Questionnaire (CTQ), a retrospective self-report of maltreatment (e.g., physical, sexual, emotional abuse, and/or severe neglect). Previous literature has highlighted discrepancies between prospective and retrospective reports of maltreatment (Baldwin et al. 2019), suggesting the two may identify separate populations with different associations to psychopathology (Baldwin et al. 2024). Thus, it is important to consider the current study’s findings within the context of prospective CM reporting. Recent work from our group, however, demonstrated agreement between CTQ measures and prospective reports of CM in the absence of lifetime SUD (Löfberg et al. 2023). Conversely, in lifetime SUD populations, disagreement between retrospective (i.e., CTQ) and prospective assessments of maltreatment emerge, suggesting that the CTQ may not perform well in this population. Thus, the present study defined CM status based on prospective documentation.

## Methods

### Overview

This study consisted of three visits: one screening visit, one behavioral laboratory session, and one Magnetic Resonance Imaging session. The present manuscript will only discuss the behavioral laboratory session, with other outcomes published elsewhere (Perini et al. 2023). During the behavior session, blood samples were collected at five-time points before and after an acute stress reactivity task. All participants completed breath and urine screens for alcohol and drugs before the laboratory session.

### Participants

Participants were recruited at Linköping University from March 2017 to July 2020. A total of 101 participants were included in the study and divided into four groups across the dimensions of CM and SUD (see Table 1). The study consisted of 4 groups recruited based on the presence or absence of CM and SUD histories: a CM + SUD group, with history of both CM and SUD; a CM only group; a SUD only clinical control group; and a healthy control group with no history of CM or SUD.

**Table 1:** Study demographics. Demographic, clinical, and psychological characteristics of the study population

Demographic	CM + SUD *N* = 28	CM only *N* = 24	SUD only *N* = 25	Healthy Control *N* = 24	*p*-value
Sex: Female	15 (54%)	17 (71%)	12 (48%)	13 (54%)	0.41
Age	28.9 (3.5)	28.9 (3.9)	27.5 (3.3)	28.3 (5.2)_	0.56
Education					<0.001
*Elementary school*	5 (18%)	1 (4%)	6 (24%)	0 (0%)	
*Vocational education*	14 (50%)	13 (54%)	11 (44%)	1 (4%)	
*High School*	4 (14%)	1 (4%)	4 (16%)	2 (8%)	
*University*	4 (14%)	7 (29%)	4 (16%)	21 (88%)	
Current psychiatric diagnosis	23 (82%)	9 (38%)	20 (80%)	1 (4%)	< 0.001
AUDIT	8.2 (5.7)	4.3 (2.6)	6.3 (6.2)	3.9 (3.4)	0.005
DUDIT	3.8 (7.4)	0.1 (0.4)	6.2 (7.9)	0.0 (0.0)	< 0.001
CPRS Depression scores (MADRS)	8.1 (4.7)	4.0 (3.7)	5.7 (4.1)	1.7 (1.8)	< 0.001
CPRS Anxiety scores	7.9 (4.9)	5.8 (3.9)	7.0 (3.6)	3.0 (2.4)	< 0.001
*Psychotropic medication*	13 (46%)	4 (17%)	15 (60%)	3 (13%)	< 0.001
Current SUD/AUD (MINI)	13 (46%)	0 (0%)	10 (40%)	0 (0%)	< 0.001
Current SUD (MINI)	6 (21%)	0 (0%)	3 (11%)	0 (0%)	0.003
Current AUD (MINI)	11 (39%)	0 (0%)	8 (28%)	0 (0%)	< 0.001
DERS total scores	43.1 (16.4)	34.8 (14.2)	40.6 (15.1)	25.9 (7.3)	< 0.001
CTQ scores					
*Total scores*	50.3 (19.8)	51.5 (18.9)	42.6 (16.1)	28.3 (3.9)	< 0.001
*Physical abuse*	9.4 (4.8)	8.7 (3.9)	6.6 (2.9)	5.1 (0.3)	< 0.001
*Sexual abuse*	8.4 (5.5)	9.8 (6.6)	5.2 (0.7)	5.0 (0.0)	< 0.001
*Emotional abuse*	12.1 (5.7)	11.6 (5.4)	10.7 (5.4)	5.7 (1.0)	< 0.001
*Physical neglect*	8.3 (3.0)	8.9 (4.4)	8.4 (4.6)	5.3 (0.7)	0.003
*Emotional neglect*	12.1 (5.7)	12.5 (4.7)	11.6 (5.5)	7.2 (2.9)	< 0.001

Data are presented as mean (SD) for continuous measures, and n (%) for categorical measures. The healthy control group drove between-group differences in sociodemographic scores. ^1^ongoing psychiatric diagnosis according to MINI screening; ^2^stable standard doses for at least three months of common psychotropic medications. *AUDIT* Alcohol Use Disorders Identification Test, *DUDIT* Drug Use Disorders Identification Test, CPRS Comprehensive Psychopathological Rating Scale, self-report, *DERS* Difficulties in Emotion Regulation Scale; *CTQ* childhood trauma questionnaire, *ADHD* total number of symptoms from the inattentive and hyperactive impulsive subscales

All included CM-exposed participants (the CM + SUD group and CM only group) had documented CM (e.g., physical, sexual abuse, and/or severe neglect) as children or adolescents and were referred to a specialized Trauma Unit within the Child & Adolescent Psychiatry Department of Linköping University Hospital. Documented lifetime SUD was identified with the regional health care register and through contact with addictions clinics in the Region of Östergötland. Current SUD diagnosis was assessed using a structured Mini International Neuropsychiatric Interview (MINI-7) (Sheehan et al. 1998) self-reports of current problems, and drug screening of urine samples. Participant characteristics are further detailed in (Perini et al. 2023).

The study was approved by the Regional Ethics Review Board in Linköping, Sweden (Dnr 2015/256 − 31, and 2017/41 − 32).

### Acute stress test

**Fig. 2:**
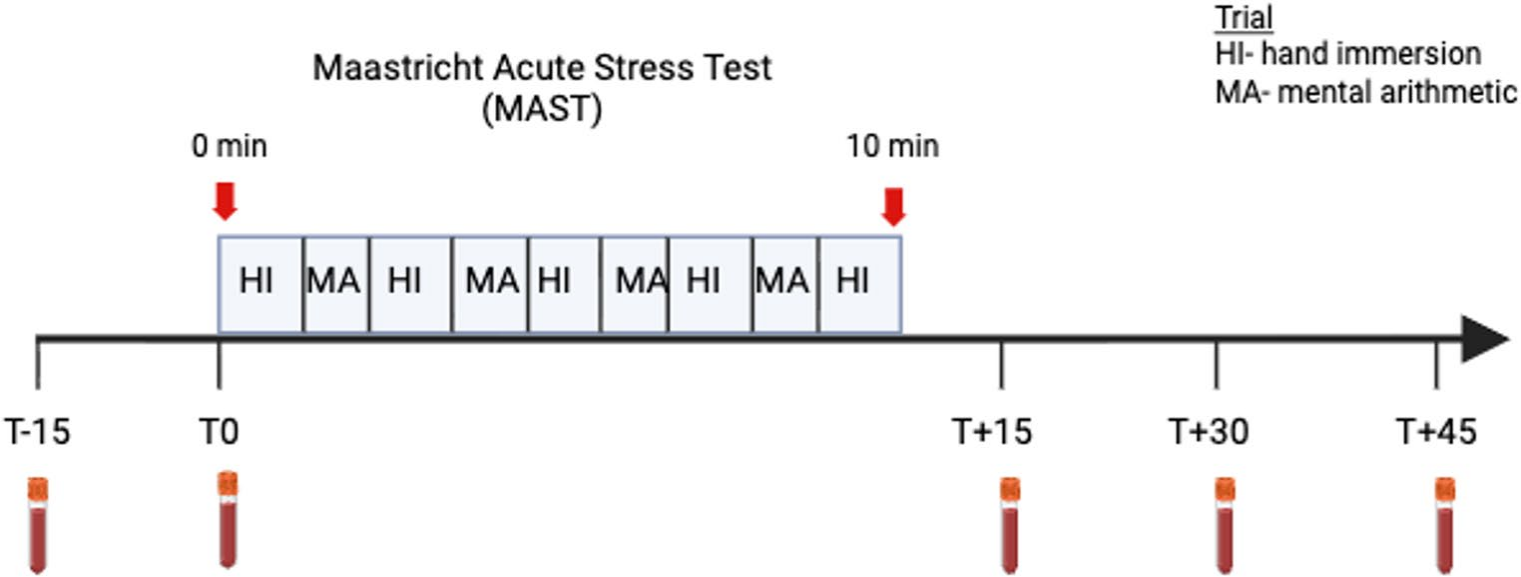
MAST paradigm. Participants alternate between HI trials lasting varying durations between 60 and 90 s, and MA trials for the 10-minute duration of the task

Upon arrival, all participants were fitted for an intravenous catheter for blood sample collection. Participants completed the Maastricht Acute Stress Test (MAST), a stress protocol combining a cold pressor test and mental arithmetic to provoke optimal autonomic and glucocorticoid stress responses (Smeets et al. 2012) (see Figure 2). The MAST requires participants to place a hand into cold water (“hand immersion” (HI) trials) maintained at a constant 2°C, for varying durations (between 60 and 90 seconds) five times across the ten-minute duration of the task. At the intervals between HI trials, participants are instructed to perform mental arithmetic (“mental arithmetic” (MA) trials) as fast and accurately as possible, receiving negative feedback when they make mistakes or instruction to speed up their response time.

Blood samples were collected at five time points relative to stress administration: −15, 0, +15, +30, and +45 min to measure baseline and stress-induced changes in peripheral KYN metabolites (Trp, KYN, KYNA, 3-hydroxykynurine (3-HK), QUIN, picolinic acid (PIC), and nicotinamide) eCBs (AEA, 2-AG, OEA, and PEA), inflammatory markers (CRP, IL-6, IL-10, and TNF), and cortisol. Samples were immediately centrifuged for 10 minutes at 4 °C, 2000 g, aliquoted, and stored at −20 degrees °C. The samples were later stored at −70°C, until analyses were conducted.

### Endocannabinoid analysis

As previously published (Stensson et al. 2017), the eCBs (AEA and 2-AG) and N-acylethanolamines (NAEs), oleoylethanolamide (OEA) and palmitoylethanolamide (PEA) were extracted and analyzed using liquid chromatography tandem mass spectrometry (LC-MS/MS).

### Inflammatory marker analysis

To extract levels of IL-6 from blood samples, the V-PLEX proinflammatory Panel 1, from the MSD Multi-spot assay system was used, according to manufacturer instructions (Meso Scale Diagnostics, LLC, 2024). The method has been validated for reliable quantification of IL-6 -levels in the range 0.633–488.633 pg/mL (Lee et al. 2006). Plasma levels of IL-10 and Tumor Necrosis Factor (TNF) were likewise extracted from blood samples using the same V-PLEX proinflammatory Panel 1 from the MSD Multi-spot assay system. Quantification of IL-10 and TNF with this method has been validated for the ranges 0.04–233.04 pg/mL and 0.004–248.004 pg/mL, respectively (Meso Scale Diagnostics, LLC, 2024).

Plasma levels of highly sensitive C-reactive protein (hsCRP) were analyzed at Clinical Chemistry in the Linköping hospital using a cobas c 504 machine (Roche Diagnostics Scandinavia AB).

### Cortisol analysis

To obtain plasma cortisol levels from the blood samples, the DetectX Cortisol Enzyme Immunoassay kit was used according to manufacturer instructions (Arbor Assays, Ann Arbor, MI).

### KYN metabolite analysis

Plasma levels of KYN and its metabolites, including KYNA, 3-HK, PIC, nicotinamide, and QUIN were quantified using ultra-performance liquid chromatography-tandem mass spectrometry, described in detail in (Trepci et al. 2020). The metabolites were detected in higher concentrations than the lowest level of quantification in all samples. Six samples were run in duplicates, and the mean coefficient of variance was less than 5%.

### Statistical analysis

All statistical analysis was conducted using IBM SPSS Statistics, version 29.0.1.1 (244). Variables that violated normality were log-transformed to meet normality criteria.

To identify the underlying structure in the data, we carried out a factor analysis. We included baseline (timepoint −15) and stress-induced (calculated as the area under the curve; AUC) changes in all variables: Trp, KYN, KYNA, 3-HK, QUIN, PIC, nicotinamide, AEA, 2-AG, OEA, PEA, CRP, IL-6, IL-10, TNF, and cortisol. Variables were normalized using Z-scoring, and missing values were replaced with the mean for that variable. We used a principal component extraction, and normalized varimax rotation. Factors were retained based on eigenvalues greater than 1. Factor scores for each subject were saved as regression variables. Differences in biomarkers with respect to CM and SUD status were assessed using univariate ANOVA with CM and SUD status included as 2-levels variables (CM +/-, SUD +/-). Tukey post hoc tests were performed on significant ANOVA outcomes.

## Results

### Characteristics of the study population

A total of 101 individuals were included in this study and were divided into four groups: a CM + SUD group (*N* = 28; 15 female, 13 male; mean age 28.9 ± 3.5); a CM only group (*N* = 24; 17 females, 7 males; mean age 28.9 ± 3.9); a SUD only group (*N* = 25; 12 females, 13 males; mean age 27.5 ± 3.3); and a healthy control group (*N* = 24; 13 females;11 males; mean age of 28.5 (+/- 5.2). For comprehensive demographics information, see Table 1.

### Baseline and stress-induced changes in biomarkers

Analysis of baseline and stress-induced changes in eCBs and cortisol levels, peripheral KYN metabolites, eCBs and related ligands, inflammatory markers, and cortisol levels have been reported elsewhere (Perini et al. 2023).

### Factor analysis

A factor analysis reduced all biomarker data into 34 factors, 9 of which met the criteria of eigenvalue greater than 1. The majority of total variance was explained by KYN metabolites and eCBs. The total variance explained by the factor analysis was 78.3%. KYN metabolites loaded onto three factors: 1,3, and 7. Factor 1 accounted for 17.9% of variance and was comprised of baseline and stress-induced changes in KYN, QUIN, 3-HK, and, to a slightly lesser extent, KYNA and baseline CRP levels. Baseline and stress-induced changes in other KYN metabolites separated onto Factor 3 (11.8% of variance), comprised of Trp, PIC, KYNA, 3-HK, and CRP, and Factor 7 (5.5% of variance), consisting of nicotinamide.

Another large portion of variance was explained by eCBS, which loaded onto two factors: 2 and 9. Factor 2 was made of baseline and stress-induced changes in AEA, OEA, and PEA, explaining 14.8% of variance. Baseline and stress-induced changes in 2-AG separated from other eCBs onto Factor 9 (3.4% of variance). For a complete overview of the factor analysis, including baseline and stress-induced changes in inflammatory markers, CRP, IL-6, IL-10, TNF, as well as cortisol, refer to Table 2.

**Table 2:**
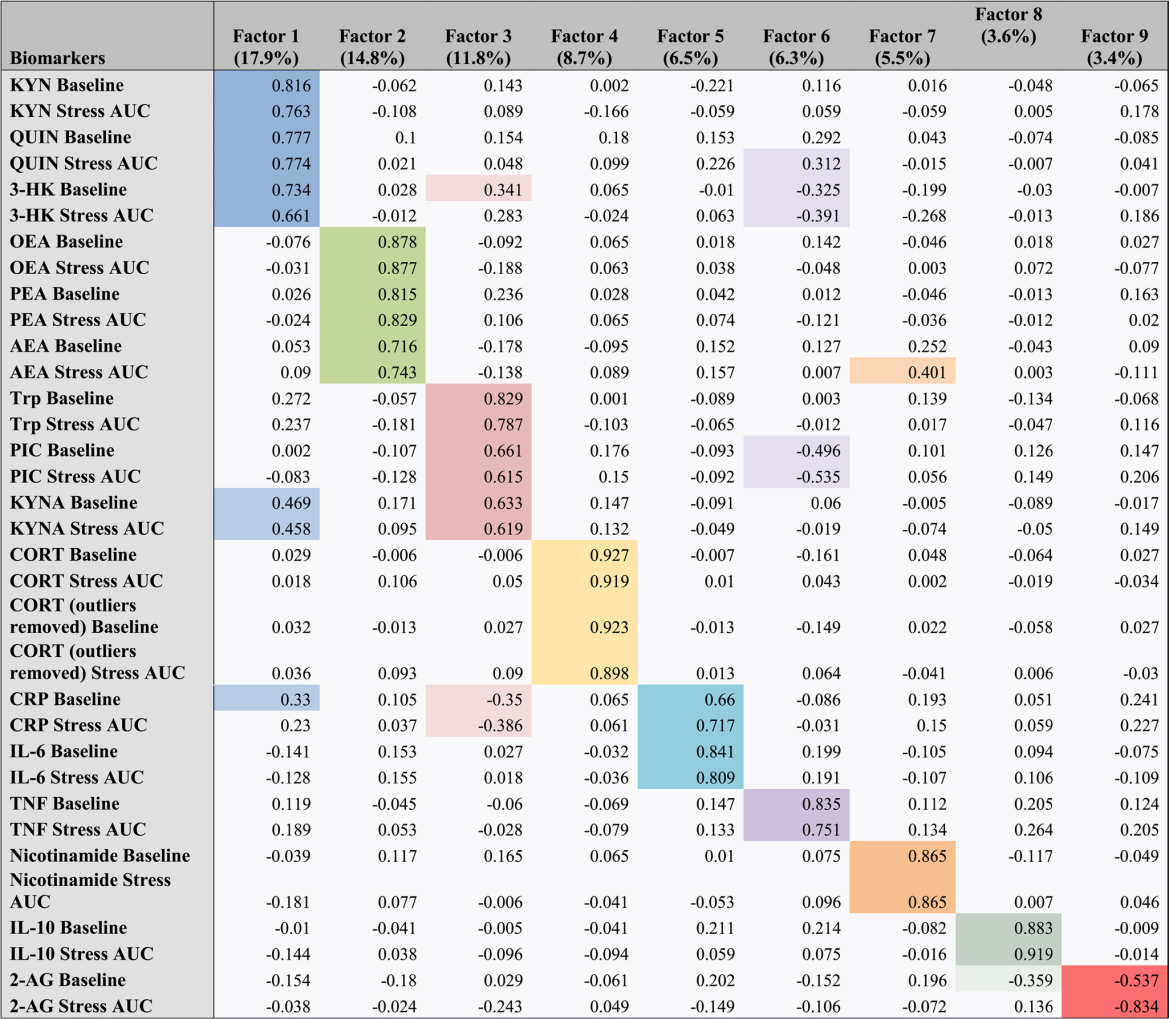
Factor analysis rotated matrix. Factor analysis reduced biomarker data into 9 factors explaining 78.3% of total variance, with the majority of variance explained by KYN pathway metabolites and eCBs

Factor 1, comprised of peripheral baseline and stress-induced changes in KYN, QUIN, 3-HK, and to a lesser extent KYNA and baseline CRP explained 17.9% of total variance, while Factor 2, comprised of peripheral baseline and stress-induced changes in AEA, OEA, and PEA explained 14.8% of variance. Colored variables within each factor represent the variables that significantly load onto the given factor. Lighter shading indicates a significant loading of the variable on the given factor when the variable has a stronger association with another factor

### Relationship between CM and SUD status on factor analysis scores

**Fig. 3:**
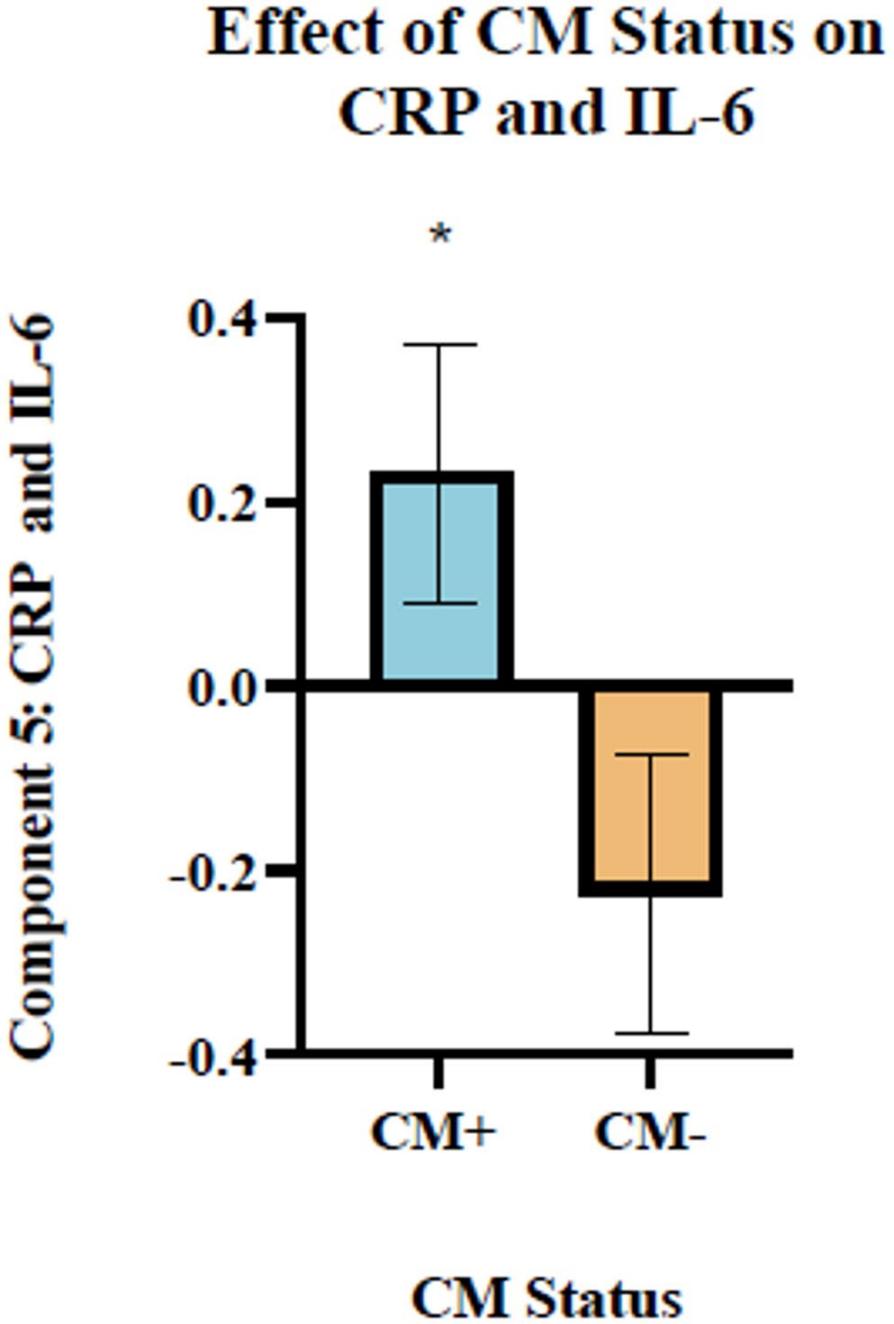
Figure 3. Factor 5, made of IL-6 and CRP, was greater in CM-exposed individuals. There was a main effect of CM exposure (CM+) on Factor 5 (F(1,103) = 4.691, p=0.033, η²p (0.044)), composed of baseline and stress-induced levels of CRP and IL-6, such that the presence of CM indicated greater levels of these variables

A subsequent 2 x 2 factor ANOVA assessing the relationship between factors generated in the factor analysis and CM and SUD status separately was conducted (results for all factors are reported in the supplementary materials). The ANOVA revealed a main effect of CM status on Factor 5, comprised of baseline and stress-induced changes in the pro-inflammatory markers IL-6 and CRP. Individuals with prospectively determined exposure to CM (CM+) had significantly higher levels of Factor 9 (F(1,103) = 4.691, p=0.033, η²p (0.044)) compared to their non-exposed counterparts (CM-; see Figure 3).

**Fig. 4:**
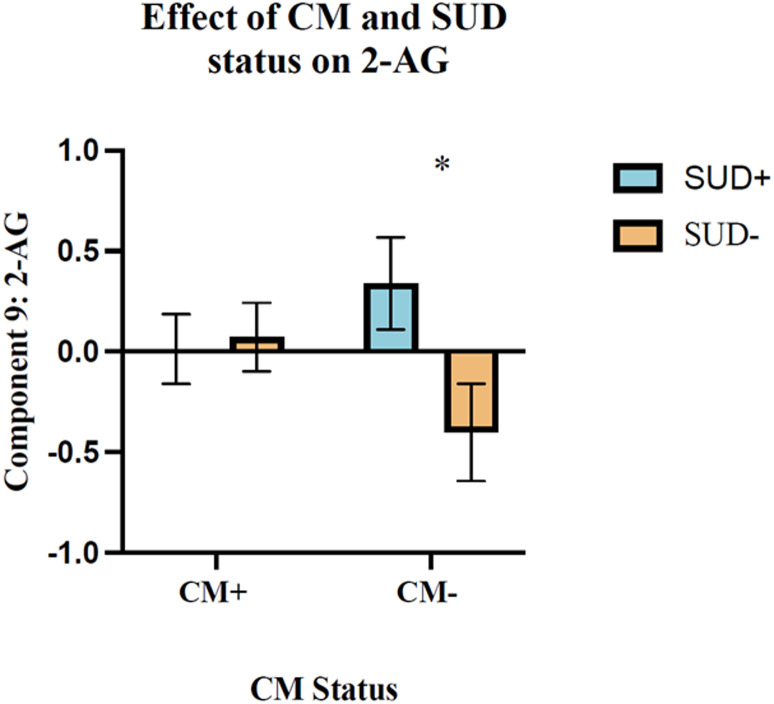
SUD and CM exposure status differentially affect the 2-AG driven Factor 9. Factor 9 was comprised of baseline and stress-induced changes in 2-AG levels. Increases in levels of Factor 9 in the absence of CM were observed in those with SUD, reflecting decreases in 2-AG (F(1,103) = 4.427, *p* = 0.038, η²p (0.041)). Conversely, reduced levels of Factor 9 in the absence of CM seen in those without SUD indicate elevated levels of 2-AG

Additionally, CM and SUD status had a significant interaction effect on Factor 9, on which baseline and stress-induced changes in 2-AG loaded negatively (F(1,103)= 4.427, p=0.038, η²p (0.041)), such that greater levels of Factor 9 reflected reductions in 2-AG (see Figure 4).

Tukey post hoc analysis identified a difference within the CM- (p=0.042), but not the CM+ groups. Specifically, individuals with no history of CM or SUD pathology (SUD-) displayed reductions of Factor 9, indicative of elevated baseline and stress-induced changes in 2-AG. Conversely, the presence of SUD (SUD+) in individuals without CM exposure was related to elevated levels of Factor 9, indicative of reductions in baseline and stress-induced changes in 2-AG.

## Discussion

Our goal was to investigate how biomarkers, including KYN metabolites, eCBs, inflammatory markers, and cortisol, relate to one another and potentially differ across groups with or without histories of CM or SUD. Beyond baseline levels of these molecules, we also included variables representative of stress-induced changes. These targets were selected based on existing literature suggesting crosstalk between these systems, particularly the KYN pathway and eCB system (Comai et al. 2024; Wences Chirino et al. 2023), as important contributors to the development of stress-related pathologies, including SUD (Morales-Puerto et al. 2021). The primary focus was a factor analysis, which reduced data from all 34 biomarkers into the 9 factors represented in Table 2. We subsequently explored the relationship between these factors and CM and SUD status. 

Overall, we found that the majority of variance was explained by KYN metabolites at baseline and in response to stress exposure. Factor 1, a KYN metabolite factor, was comprised of baseline and stress-induced changes in KYN, QUIN, 3-HK, KYNA, as well as baseline CRP levels. Notably, the KYN metabolites that were most highly associated with Factor 1 are of the “inflammatory” or “neurotoxic” branch of the KYN pathway. For instance, 3-HK and QUIN have been shown to exhibit neurotoxic properties, including excitotoxicity (Guidetti and Schwarcz 1999; Lugo-Huitrón et al. 2013; Schwarcz et al. 1983; Schwarcz and Köhler 1983; Stone and Perkins 1981), and elevations in these metabolites have been observed in various psychiatric and degenerative disorders (Leclercq et al. 2021; Schwarcz et al. 2012). Similarly, recent research in human populations has implicated enhanced levels of KYN in mood disorders, such as depression (Comai et al. 2024) and SUD pathology, with the majority of the SUD studies conducted in alcohol use disorder populations (Jang et al. 2022; Morales-Puerto et al. 2021; Wang et al. 2023). The strong association of these neurotoxic metabolites with Factor 1 may additionally explain the loading of baseline CRP levels on this factor, as shifts in Trp metabolism along the KYN pathway are promoted by inflammation (O’Connor et al. 2009; Yoshida et al. 1979; Yoshida and Hayaishi 1978). Interestingly, the neuroprotective metabolite KYNA also loaded onto Factor 1 to a smaller but significant extent, somewhat complicating our interpretation of this factor. However, previous studies have reported elevations in KYNA in response to a stressor (Chiappelli et al. 2018; Herhaus et al. 2021), potentially related to heightened KYN production and metabolism during stress. Upregulated KYN production and metabolism contributes to increased activity along both the neuroprotective and neurotoxic branches of the KYN pathway and may, in part, explain the grouping of KYNA with neurotoxic KYN metabolites within our factor analysis.

A second KYN metabolite factor, Factor 3, also explained a significant portion of total variance. Factor 3 was comprised of baseline and stress-induced changes in Trp, KYNA, PIC, 3-HK. Distinct from Factor 1, the neuroprotective KYN metabolites were the most strongly associated with this factor. These included KYNA and PIC, which previous studies have demonstrated to have neuroprotective properties, potentially counteracting the toxic effects of QUIN (Beninger et al. 1994; Foster et al. 1984). Baseline and stress-induced changes in CRP levels additionally loaded onto this factor and displayed a negative relationship with these neuroprotective KYN metabolites. This grouping may reflect the relationship between neuroprotective KYN factors and an “anti-inflammatory” state. Interestingly, 3-HK at baseline and throughout stress exposure also loaded onto Factor 3. The balance between neurotoxic and neuroprotective KYN metabolites, as well as their relationship to inflammatory signaling, has been proposed as a potential mechanism underlying susceptibility to various psychiatric disorders and suicidality (Brundin et al. 2016; Marx et al. 2021). The present analysis found neurotoxic (i.e., QUIN, 3-HK) and neuroprotective (i.e., KYNA, PIC) KYN metabolites to partially group together. Several studies have reported KYN pathway dysfunction in AUD and SUD. However, our results did not find that stress-induced KYN pathway changes were moderated by SUD or CM status, suggesting that the KYN pathway may be involved in other, non-stress-related aspects of these disorders. Future studies are needed to more clearly elucidate the role of the KYN pathway in CM and SUD.

In addition to KYN metabolites, eCBs also accounted for a significant portion of the total variance. Specifically, Factor 2 was comprised of AEA, OEA, and PEA at baseline and across stress exposure. Baseline and stress-induced changes in 2-AG separated from other eCBs and loaded onto Factor 9. Furthermore, inflammatory markers separated onto three factors. Factor 5, a pro-inflammatory factor, was comprised of baseline and stress-induced changes in CRP and IL-6. Interestingly, levels of TNF at baseline and following stress exposure loaded onto Factor t 6 with various KYN metabolites. Baseline and stress-induced changes in levels of IL-10 loaded onto Factor 8 and were inversely related to baseline 2-AG levels. Stress-induced changes and baseline levels of 2-AG loaded onto Factor 9. Cortisol levels at baseline and across stress exposure loaded onto Factor 4, separating from other biomarkers.

We found a main effect of CM exposure on Factor 5, a pro-inflammatory factor comprised of baseline and stress-induced changes in CRP and IL-6. Specifically, individuals with a history of CM exhibited higher levels of Factor 5 than their non-exposed counterparts. Our findings align with the robustly established relationship between exposure to chronic stressors, such as CM, and enhanced pro-inflammatory signaling. Meta-analyses assessing immune signaling in CM populations have found that CM exposure significantly increases baseline peripheral levels of CRP, IL-6, and TNF (Baumeister et al. 2016; Brown et al. 2021). Likewise, longitudinal studies prospectively assessing CM status have found adults with a history of maltreatment to display elevated baseline levels of CRP independent of co-occurring early life risks (including low birth weight, socioeconomic disadvantage, and low intelligence quotient), adulthood stress, and adult health and health behaviors (Danese et al. 2007, 2008). Previous work has additionally demonstrated enhanced pro-inflammatory signaling in CM populations in response to an acute laboratory stressor. Individuals with a history of CM exhibit enhanced reactivity of IL-6 during psychosocial stress tests compared to healthy controls (Carpenter et al. 2010; Schreier et al. 2020). Our findings support the positive relationship between CM exposure and baseline and stress-induced pro-inflammatory signaling, particularly of CRP and IL-6.

We also found a difference relative to CM and SUD status on Factor 9, an eCB factor. Baseline and stress-induced changes in levels of the eCB ligand 2-AG were negatively associated with Factor 9. In the absence of CM (CM-), levels of Factor 9 were significantly increased if SUD pathology was present (SUD+), reflecting reductions in baseline and stress-induced changes in 2-AG levels. Conversely, when both CM exposure (CM-) and SUD pathology were absent (SUD-), decreases in Factor 9 and thereby enhancements of 2-AG were observed. As a phasic regulator of stress, 2-AG increases in response to acute stressors, adaptively blunting the magnitude and duration of the stress response (Hill et al. 2010, 2011; Wang et al. 2012). Exposure to chronic stressors such as CM, however, are associated with dysregulated eCB signaling and modulation of the different arms of stress response, including the HPA axis (Hill et al. 2009; Patel et al. 2009; Rossi et al. 2008; Wamsteeker et al. 2010). Consequently, CM-induced alterations of eCB signaling may contribute to the development of stress-related disorders such as SUD (Fuentes et al. 2024).

In the present investigation, baseline and stress-induced changes in 2-AG were reduced in the presence of SUD (SUD+) in individuals without CM (CM-), indicated by greater levels of Factor 9. These findings may therefore suggest a mediating role of 2-AG signaling in SUD pathology. This theory is supported by recent preclinical work demonstrating pharmacological enhancement of 2-AG via inhibition of its primary degrading enzyme, monoacylglycerol lipase, attenuates the negative withdrawal effects of ethanol (Morgan et al. 2022). Interestingly, pharmacological inhibition of DAGL, the primary synthesizing enzyme of 2-AG, which results in reduced levels of 2-AG was found to reduce alcohol consumption in a variety of mouse models (Winters et al. 2021). Taken together, 2-AG signaling appears to be implicated in different aspects of SUD pathology, specifically in models of alcohol use disorder (for a comprehensive review see (Elliott et al. 2025). The present findings expand upon this work, demonstrating an interaction effect between CM and SUD status, not limited to AUD, on 2-AG signaling in humans. Interestingly, in the presence of CM (CM+), no effect of SUD status was observed on Factor 9. It is possible that CM may play an interactive role in the relationship between SUD and 2-AG signaling. However, due to the cross-sectional design of the present study, mechanistic conclusions cannot be drawn. Future research should further examine the nature of this relationship to understand the mechanistic role of 2-AG signaling in SUD pathology and CM populations.

We did not, however, observe a significant impact of CM or SUD status on the KYN metabolite factors, contrasting existing research demonstrating an association between KYN pathway metabolism, chronic stress exposure, and stress-related psychopathologies such as SUD (Comai et al. 2024; Contella et al. 2024; Leclercq et al. 2021; Wences Chirino et al. 2023) as discussed earlier. However, research translating the impact of chronic stress on KYN metabolic function in humans is generally lacking. Thus, our more complex population, including both current or lifetime SUD diagnosis and CM history, may contribute to the differences in results. Specifically, the current project included participants with both previous and ongoing SUD, which may have unique biomarker profiles and contribute to the discrepancy between our results and previous findings. The present study additionally assessed CM exposure prospectively, which may explain why our findings do not validate previous work in retrospectively assessed CM populations (Löfberg et al. 2023). Furthermore, while previous work in healthy humans has utilized the MAST to assess stress responsivity of KYN metabolites (La Torre et al. 2021), the temporal response of KYN metabolites to acute stressors may be altered in clinical populations. Thus, the current stress paradigm may not have been able to capture stress-induced changes in KYN metabolites and thereby the effect of CM and SUD status on KYN metabolite-driven factors.

Our study has several limitations. While our sample size was considerable overall (*N* = 101), the 2 × 2 factorial design resulted in small subgroups (n = ~ 25), limiting our statistical power. Furthermore, we were underpowered to assess the contributions of CM subtype. Additionally, due to the prospective assessment of CM status in the study population, the current findings are limited in their generalizability to retrospectively assessed CM populations (Baldwin et al. 2024). Furthermore, the clinical control “SUD-only” group, comprised of individuals with current or lifetime SUD diagnosis but no prospectively documented CM, retrospectively reported CM exposure on the CTQ at rates comparable to the CM groups. While the CTQ has been shown to be a limited measure of maltreatment in SUD populations (Löfberg et al. 2023), self-reported CM exposure in our “SUD-only” group nuances the interpretation of our results.

Potential confounding effects of psychiatric comorbidities and medications should also be considered in the interpretation of the current results. However, participants prescribed psychotropic medications were required to have stable use for at least three months to be included in the study, and diagnoses of current or lifetime schizophrenia, bipolar or psychotic disorder, or current suicidality were excluded. The present analyses did not investigate potential confounding effects of other comorbid psychiatric conditions, which should be considered for future investigations, as KYN metabolites, eCBs, stress, and inflammatory biomarkers are impacted in various psychiatric diagnoses.

Finally, the cross-sectional approach means we are unable to determine if changes in the biomarkers reported are a consequence of exposure or representative of inherent differences existing prior to exposure. Regardless, we provide novel insights into the relationship between a variety of baseline and stress-induced biomarkers and have pro-inflammatory biomarkers, specifically CRP and IL-6, and 2-AG signaling as potential avenues worth further exploring to better understand the mechanistic underpinnings of vulnerability to SUD development in CM populations.

## Supplementary Information

Below is the link to the electronic supplementary material.

Supplementary Material 1 (DOCX 25.3 KB)

## Data Availability

The data published here is available upon request to the corresponding author.

## Abbreviations

AEA: Anandamide
CM: Childhood maltreatment
CORT: Cortisol
CRP: C reactive protein
CTQ: Childhood Trauma Questionnaire
eCB: Endocannabinoid
KYN: Kynurenine
KYNA: Kynurenic acid
QUIN: Quinolinic acid
HPA: Hypothalamus-pituitary-adrenal
HI: Hand immersion
IDO: Indoleamine 2,3-dioxygenase
IL-6: Interleukin-6
IL-10: Interleukin-10
MA: Mental arithmetic
MAST: Maastricht Acute Stress Test
OEA: Oleoylethanolamide
PEA: Palmitoylethanolamide
PIC: Picolinic acid
SUD: Substance use disorder
TDO: Trp 2,3-dioxygenase
TNF: Tumor necrosis factor
Trp: Tryptophan
2-AG: 2-Arachidonoylglycerol
3-HK: 3-hydroxykynurenine

## References

[CR1] Agarwal K, Manza P, Chapman M, Nawal N, Biesecker E, McPherson K, Dennis E, Johnson A, Volkow ND, Joseph PV (2022) Inflammatory markers in substance use and mood disorders: a neuroimaging perspective. Front Psychiatry. 10.3389/fpsyt.2022.863734 35558424 10.3389/fpsyt.2022.863734 PMC9086785

[CR2] Almogi-Hazan O, Or R (2020) Cannabis, the endocannabinoid system and immunity—the journey from the bedside to the bench and back. Int J Mol Sci 21(12):4448. 10.3390/ijms21124448 32585801 10.3390/ijms21124448 PMC7352399

[CR3] Anda RF, Felitti VJ, Bremner JD, Walker JD, Whitfield C, Perry BD, Dube SR, Giles WH (2006) The enduring effects of abuse and related adverse experiences in childhood. Eur Arch Psychiatry Clin NeuroSci 256(3):174–186. 10.1007/s00406-005-0624-4 16311898 10.1007/s00406-005-0624-4 PMC3232061

[CR4] Baldwin JR, Reuben A, Newbury JB, Danese A (2019) Agreement between prospective and retrospective measures of childhood maltreatment: a systematic review and meta-analysis. JAMA Psychiatr 76(6):584–593. 10.1001/jamapsychiatry.2019.0097 10.1001/jamapsychiatry.2019.0097 PMC6551848 30892562

[CR5] Baldwin JR, Coleman O, Francis ER, Danese A (2024) Prospective and retrospective measures of child maltreatment and their association with psychopathology: a systematic review and meta-analysis. JAMA Psychiatr 81(8):769–781. 10.1001/jamapsychiatry.2024.0818 10.1001/jamapsychiatry.2024.0818 PMC11063927 38691376

[CR6] Balsevich G, Petrie GN, Hill MN (2017) Endocannabinoids: effectors of glucocorticoid signaling. Front Neuroendocrinol 47:86–108. 10.1016/j.yfrne.2017.07.005 28739508 10.1016/j.yfrne.2017.07.005

[CR7] Baumeister D, Akhtar R, Ciufolini S, Pariante CM, Mondelli V (2016) Childhood trauma and adulthood inflammation: a meta-analysis of peripheral C-reactive protein, interleukin-6 and tumour necrosis factor-α. Mol Psychiatry 21(5):642–649. 10.1038/mp.2015.67 26033244 10.1038/mp.2015.67 PMC4564950

[CR8] Beggiato S, Ieraci A, Zuccarini M, Di Iorio P, Schwarcz R, Ferraro L (2022) Alterations in rat prefrontal cortex kynurenic acid levels are involved in the enduring cognitive dysfunctions induced by tetrahydrocannabinol exposure during the adolescence. Front Psychiatry. 10.3389/fpsyt.2022.996406 36483135 10.3389/fpsyt.2022.996406 PMC9722723

[CR9] Beninger RJ, Colton AM, Ingles JL, Jhamandas K, Boegman RJ (1994) Picolinic acid blocks the neurotoxic but not the neuroexcitant properties of quinolinic acid in the rat brain: evidence from turning behaviour and tyrosine hydroxylase immunohistochemistry. Neuroscience 61(3):603–612. 10.1016/0306-4522(94)90438-3 7969932 10.1016/0306-4522(94)90438-3

[CR10] Bilel S, Corli G, Tiziani E, Chirenti D, Dall’Acqua S, Comai S, Ferraro L, Marti M, Beggiato S (2025) Kynurenine amplifies tetrahydrocannabinol-induced sensorimotor impairment and classic tetrad effects in mice. Prog Neuropsychopharmacol Biol Psychiatry 138:111342. 10.1016/j.pnpbp.2025.111342 40139338 10.1016/j.pnpbp.2025.111342

[CR11] Brown M, Worrell C, Pariante CM (2021) Inflammation and early life stress: an updated review of childhood trauma and inflammatory markers in adulthood. Pharmacol Biochem Behav 211:173291. 10.1016/j.pbb.2021.173291 34695507 10.1016/j.pbb.2021.173291

[CR12] Brundin L, Sellgren CM, Lim CK, Grit J, Pålsson E, Landén M, Samuelsson M, Lundgren K, Brundin P, Fuchs D, Postolache TT, Traskman-Bendz L, Guillemin GJ, Erhardt S (2016) An enzyme in the kynurenine pathway that governs vulnerability to suicidal behavior by regulating excitotoxicity and neuroinflammation. Transl Psychiatry 6(8):e865. 10.1038/tp.2016.133 27483383 10.1038/tp.2016.133 PMC5022080

[CR13] Capusan AJ, Gustafsson PA, Kuja-Halkola R, Igelström K, Mayo LM, Heilig M (2021) Re-examining the link between childhood maltreatment and substance use disorder: a prospective, genetically informative study. Mol Psychiatry 26(7):3201–3209. 10.1038/s41380-021-01071-8 33824431 10.1038/s41380-021-01071-8

[CR14] Carpenter LL, Gawuga CE, Tyrka AR, Lee JK, Anderson GM, Price LH (2010) Association between plasma IL-6 response to acute stress and early-life adversity in healthy adults. Neuropsychopharmacology 35(13):2617–2623. 10.1038/npp.2010.159 20881945 10.1038/npp.2010.159 PMC2978751

[CR15] Chiappelli J, Rowland LM, Notarangelo FM, Wijtenburg SA, Thomas MAR, Pocivavsek A, Jones A, Wisner K, Kochunov P, Schwarcz R, Hong LE (2018) Salivary kynurenic acid response to psychological stress: inverse relationship to cortical glutamate in schizophrenia. Neuropsychopharmacology 43(8):1706–1711. 10.1038/s41386-018-0072-2 29728648 10.1038/s41386-018-0072-2 PMC6006286

[CR16] Choi DW (1987) Ionic dependence of glutamate neurotoxicity. J Neurosci 7(2):369–379. 10.1523/JNEUROSCI.07-02-00369.1987 2880938 10.1523/JNEUROSCI.07-02-00369.1987 PMC6568907

[CR17] Cicchetti D, Rogosch FA (2001) The impact of child maltreatment and psychopathology on neuroendocrine functioning. Dev Psychopathol 13(4):783–804 11771908

[CR18] Coelho R, Viola TW, Walss-Bass C, Brietzke E, Grassi-Oliveira R (2014) Childhood maltreatment and inflammatory markers: a systematic review. Acta Psychiatr Scand 129(3):180–192. 10.1111/acps.12217 24205846 10.1111/acps.12217

[CR19] Comai S, Bertazzo A, Brughera M, Crotti S (2020) Tryptophan in health and disease. Adv Clin Chem 95:165–218. 10.1016/bs.acc.2019.08.005 32122523 10.1016/bs.acc.2019.08.005

[CR20] Comai S, Nunez N, Atkin T, Ghabrash MF, Zakarian R, Fielding A, Saint-Laurent M, Low N, Sauber G, Ragazzi E, Hillard CJ, Gobbi G (2024) Dysfunction in endocannabinoids, palmitoylethanolamide, and degradation of tryptophan into kynurenine in individuals with depressive symptoms. BMC Med 22:33. 10.1186/s12916-024-03248-8 38273283 10.1186/s12916-024-03248-8 PMC10809514

[CR21] Contella L, Farrell CL, Boccuto L, Litwin AH, Snyder ML (2024) Impact of substance use disorder on tryptophan metabolism through the kynurenine pathway: a narrative review. Metabolites 14(11):611. 10.3390/metabo14110611 39590847 10.3390/metabo14110611 PMC11597030

[CR22] Danese A, McEwen BS (2012) Adverse childhood experiences, allostasis, allostatic load, and age-related disease. Physiol Behav 106(1):29–39. 10.1016/j.physbeh.2011.08.019 21888923 10.1016/j.physbeh.2011.08.019

[CR23] Danese A, Pariante CM, Caspi A, Taylor A, Poulton R (2007) Childhood maltreatment predicts adult inflammation in a life-course study. Proc Natl Acad Sci U S A 104(4):1319–1324. 10.1073/pnas.0610362104 17229839 10.1073/pnas.0610362104 PMC1783123

[CR24] Danese A, Moffitt TE, Pariante CM, Ambler A, Poulton R, Caspi A (2008) Elevated inflammation levels in depressed adults with a history of childhood maltreatment. Arch Gen Psychiatry 65(4):409–415. 10.1001/archpsyc.65.4.409 18391129 10.1001/archpsyc.65.4.409 PMC2923056

[CR25] Dube SR, Miller JW, Brown DW, Giles WH, Felitti VJ, Dong M, Anda RF (2006) Adverse childhood experiences and the association with ever using alcohol and initiating alcohol use during adolescence. J Adolesc Health: Official Publication Soc Adolesc Med 38(4):444e1–44410. 10.1016/j.jadohealth.2005.06.006 10.1016/j.jadohealth.2005.06.006 16549308

[CR26] Elliott GO, Petrie GN, Kroll SL, Roche DJO, Mayo LM (2025) Changes in peripheral endocannabinoid levels in substance use disorders: a review of clinical evidence. Am J Drug Alcohol Abus 51(2):152–164. 10.1080/00952990.2025.2456499 10.1080/00952990.2025.2456499 40197861

[CR27] Enoch M-A (2011) The role of early life stress as a predictor for alcohol and drug dependence. Psychopharmacology 214(1):17–31. 10.1007/s00213-010-1916-6 20596857 10.1007/s00213-010-1916-6 PMC3005022

[CR28] Foster AC, Vezzani A, French ED, Schwarcz R (1984) Kynurenic acid blocks neurotoxicity and seizures induced in rats by the related brain metabolite quinolinic acid. Neurosci Lett 48(3):273–278. 10.1016/0304-3940(84)90050-8 6237279 10.1016/0304-3940(84)90050-8

[CR29] Fuentes JJ, Mayans J, Guarro M, Canosa I, Mestre-Pintó JI, Fonseca F, Torrens M (2024) Peripheral endocannabinoids in major depressive disorder and alcohol use disorder: a systematic review. BMC Psychiatry 24:551. 10.1186/s12888-024-05986-8 39118031 10.1186/s12888-024-05986-8 PMC11308641

[CR30] Giménez-Gómez P, Pérez-Hernández M, Gutiérrez-López MD, Vidal R, Abuin-Martínez C, O’Shea E, Colado MI (2018) Increasing kynurenine brain levels reduces ethanol consumption in mice by inhibiting dopamine release in nucleus accumbens. Neuropharmacology 135:581–591. 10.1016/j.neuropharm.2018.04.016 29705534 10.1016/j.neuropharm.2018.04.016

[CR31] Guastaferro K, Shipe SL (2023) Child maltreatment types by age: implications for prevention. Int J Environ Res Public Health 21(1):20. 10.3390/ijerph21010020 38248485 10.3390/ijerph21010020 PMC10815835

[CR32] Guidetti P, Schwarcz R (1999) 3-hydroxykynurenine potentiates quinolinate but not NMDA toxicity in the rat striatum. Eur J Neurosci 11(11):3857–3863. 10.1046/j.1460-9568.1999.00806.x 10583474 10.1046/j.1460-9568.1999.00806.x

[CR33] Herhaus B, Joisten N, Wessels I, Zimmer P, Petrowski K (2021) How acute physical and psychological stress differentially influence the kynurenine pathway: a randomized cross-over trial. Psychoneuroendocrinology 134:105433. 10.1016/j.psyneuen.2021.105433 34695711 10.1016/j.psyneuen.2021.105433

[CR34] Hestad K, Alexander J, Rootwelt H, Aaseth JO (2022) The role of tryptophan dysmetabolism and quinolinic acid in depressive and neurodegenerative diseases. Biomolecules 12(7):998. 10.3390/biom12070998 35883554 10.3390/biom12070998 PMC9313172

[CR35] Hill MN, Hunter RG, McEwen BS (2009) Chronic stress differentially regulates cannabinoid CB1 receptor binding in distinct hippocampal subfields. Eur J Pharmacol 614(1–3):66–69. 10.1016/j.ejphar.2009.04.048 19426726 10.1016/j.ejphar.2009.04.048 PMC2746437

[CR36] Hill MN, McLaughlin RJ, Bingham B, Shrestha L, Lee TTY, Gray JM, Hillard CJ, Gorzalka BB, Viau V (2010) Endogenous cannabinoid signaling is essential for stress adaptation. Proc Natl Acad Sci USA 107(20):9406–9411. 10.1073/pnas.0914661107 20439721 10.1073/pnas.0914661107 PMC2889099

[CR37] Hill MN, McLaughlin RJ, Pan B, Fitzgerald ML, Roberts CJ, Lee TT-Y, Karatsoreos IN, Mackie K, Viau V, Pickel VM, McEwen BS, Liu Q, Gorzalka BB, Hillard CJ (2011) Recruitment of prefrontal cortical endocannabinoid signaling by glucocorticoids contributes to termination of the stress response. J Neurosci 31(29):10506–10515. 10.1523/JNEUROSCI.0496-11.2011 21775596 10.1523/JNEUROSCI.0496-11.2011 PMC3179266

[CR38] Huang M-C, Schwandt ML, Ramchandani VA, George DT, Heilig M (2012) Impact of multiple types of childhood trauma exposure on risk of psychiatric comorbidity among alcoholic inpatients. Alcohol Clin Exp Res 36(6):1099–1107. 10.1111/j.1530-0277.2011.01695.x 22420670 10.1111/j.1530-0277.2011.01695.x PMC3370064

[CR39] Hyman SM, Paliwal P, Chaplin TM, Mazure CM, Rounsaville BJ, Sinha R (2008) Severity of childhood trauma is predictive of cocaine relapse outcomes in women but not men. Drug Alcohol Depend 92(1–3):208–216. 10.1016/j.drugalcdep.2007.08.006 17900822 10.1016/j.drugalcdep.2007.08.006 PMC2233653

[CR40] Jang JH, Yoo SY, Park YE, Ji M-J, Park H-M, Back JH, Lee JY, Kim DJ, Lee JE, Choi J-S (2022) The kynurenine pathway and mediating role of stress in addictive disorders: a focus on alcohol use disorder and internet gaming disorder. Front Pharmacol. 10.3389/fphar.2022.865576 35479326 10.3389/fphar.2022.865576 PMC9037037

[CR41] Justinova Z, Mascia P, Wu H-Q, Secci ME, Redhi GH, Panlilio LV, Scherma M, Barnes C, Parashos A, Zara T, Fratta W, Solinas M, Pistis M, Bergman J, Kangas BD, Ferré S, Tanda G, Schwarcz R, Goldberg SR (2013) Reducing cannabinoid abuse and preventing relapse by enhancing endogenous brain levels of kynurenic acid. Nat Neurosci 16(11):1652–1661. 10.1038/nn.3540 24121737 10.1038/nn.3540 PMC3835353

[CR42] La Torre D, Dalile B, de Loor H, Van Oudenhove L, Verbeke K (2021) Changes in kynurenine pathway metabolites after acute psychosocial stress in healthy males: a single-arm pilot study. Stress (Amsterdam Neth) 24(6):920–930. 10.1080/10253890.2021.1959546 10.1080/10253890.2021.1959546 34320918

[CR43] Lashgari N-A, Roudsari NM, Shayan M, Niazi Shahraki F, hosseini Y, Momtaz S, Abdolghaffari AH (2023) IDO/kynurenine; novel insight for treatment of inflammatory diseases. Cytokine 166:156206. 10.1016/j.cyto.2023.156206 37120946 10.1016/j.cyto.2023.156206

[CR44] Leclercq S, Schwarz M, Delzenne NM, Stärkel P, de Timary P (2021) Alterations of kynurenine pathway in alcohol use disorder and abstinence: a link with gut microbiota, peripheral inflammation and psychological symptoms. Transl Psychiatry 11:503. 10.1038/s41398-021-01610-5 34599147 10.1038/s41398-021-01610-5 PMC8486842

[CR45] Lee JW, Devanarayan V, Barrett YC, Weiner R, Allinson J, Fountain S, Keller S, Weinryb I, Green M, Duan L, Rogers JA, Millham R, O’Brien PJ, Sailstad J, Khan M, Ray C, Wagner JA (2006) Fit-for-Purpose method development and validation for successful biomarker measurement. Pharm Res 23(2):312–328. 10.1007/s11095-005-9045-3 16397743 10.1007/s11095-005-9045-3

[CR46] Lijffijt M, Hu K, Swann AC (2014) Stress modulates Illness-Course of substance use disorders: A translational review. Front Psychiatry 5. 10.3389/fpsyt.2014.00083 10.3389/fpsyt.2014.00083 PMC4101973 25101007

[CR47] Löfberg A, Gustafsson PA, Gauffin E, Perini I, Heilig M, Capusan AJ (2023) Assessing childhood maltreatment exposure in patients without and with a diagnosis of substance use disorder. J Addict Med 17(3):263–270. 10.1097/ADM.0000000000001091 37267165 10.1097/ADM.0000000000001091 PMC10241443

[CR48] Lugo-Huitrón R, Ugalde Muñiz P, Pineda B, Pedraza-Chaverrí J, Ríos C, Pérez-de la Cruz V (2013) Quinolinic acid: an endogenous neurotoxin with multiple targets. Oxidative Med Cell Longev 2013:104024. 10.1155/2013/104024 10.1155/2013/104024 PMC3780648 24089628

[CR49] MacMillan HL, Georgiades K, Duku EK, Shea A, Steiner M, Niec A, Tanaka M, Gensey S, Spree S, Vella E, Walsh CA, De Bellis MD, Van der Meulen J, Boyle MH, Schmidt LA (2009) Cortisol response to stress in female youths exposed to childhood maltreatment: results of the youth mood project. Biol Psychiatry 66(1):62–68. 10.1016/j.biopsych.2008.12.014 19217075 10.1016/j.biopsych.2008.12.014 PMC3816014

[CR50] Marques-Feixa L, Palma-Gudiel H, Romero S, Moya-Higueras J, Rapado-Castro M, Castro-Quintas Á, Zorrilla I, José Muñoz M, Ramírez M, Mayoral M, Mas A, José Lobato M, Blasco-Fontecilla H, Fañanás L, EPI-Young Stress GROUP (2023) Childhood maltreatment disrupts HPA-axis activity under basal and stress conditions in a dose-response relationship in children and adolescents. Psychol Med 53(3):1060–1073. 10.1017/S003329172100249X 34269169 10.1017/S003329172100249X PMC9976019

[CR51] Marx W, McGuinness AJ, Rocks T, Ruusunen A, Cleminson J, Walker AJ, Gomes-da-Costa S, Lane M, Sanches M, Diaz AP, Tseng P-T, Lin P-Y, Berk M, Clarke G, O’Neil A, Jacka F, Stubbs B, Carvalho AF, Quevedo J, Fernandes BS (2021) The kynurenine pathway in major depressive disorder, bipolar disorder, and schizophrenia: a meta-analysis of 101 studies. Mol Psychiatry 26(8):4158–4178. 10.1038/s41380-020-00951-9 33230205 10.1038/s41380-020-00951-9

[CR52] Mayo LM, Asratian A, Lindé J, Holm L, Nätt D, Augier G, Stensson N, Vecchiarelli HA, Balsevich G, Aukema RJ, Ghafouri B, Spagnolo PA, Lee FS, Hill MN, Heilig M (2020) Protective effects of elevated anandamide on stress and fear-related behaviors: translational evidence from humans and mice. Mol Psychiatry 25(5):993–1005. 10.1038/s41380-018-0215-1 30120421 10.1038/s41380-018-0215-1

[CR53] Meso Scale Discovery. (2024). Retrieved November 2, 2025. https://www.mesoscale.com/

[CR54] Micale V, Drago F (2018) Endocannabinoid system, stress and HPA axis. Eur J Pharmacol 834:230–239. 10.1016/j.ejphar.2018.07.039 30036537 10.1016/j.ejphar.2018.07.039

[CR55] Morales-Puerto N, Giménez-Gómez P, Pérez-Hernández M, Abuin-Martínez C, de Biedma-Elduayen G, Vidal L, Gutiérrez-López R, O’Shea MD, E., Colado MI (2021) Addiction and the kynurenine pathway: A new dancing couple? Pharmacology Therapeutics 223:107807. 10.1016/j.pharmthera.2021.107807 33476641 10.1016/j.pharmthera.2021.107807

[CR56] Morena M, Patel S, Bains JS, Hill MN (2016) Neurobiological interactions between stress and the endocannabinoid system. Neuropsychopharmacology 41(1):80–102. 10.1038/npp.2015.166 26068727 10.1038/npp.2015.166 PMC4677118

[CR57] Morgan A, Adank D, Johnson K, Butler E, Patel S (2022) 2‐arachidonoylglycerol‐mediated endocannabinoid signaling modulates mechanical hypersensitivity associated with alcohol withdrawal in mice. Alcohol Clin Exp Res 46(11):2010–2024. 10.1111/acer.14949 36125319 10.1111/acer.14949 PMC10091740

[CR58] O’Connor JC, Lawson MA, André C, Moreau M, Lestage J, Castanon N, Kelley KW, Dantzer R (2009) Lipopolysaccharide-induced depressive-like behavior is mediated by indoleamine 2,3-dioxygenase activation in mice. Mol Psychiatry 14(5):511–522. 10.1038/sj.mp.4002148 18195714 10.1038/sj.mp.4002148 PMC2683474

[CR59] Patel S, Kingsley PJ, Mackie K, Marnett LJ, Winder DG (2009) Repeated homotypic stress elevates 2-arachidonoylglycerol levels and enhances short-term endocannabinoid signaling at inhibitory synapses in basolateral amygdala. Neuropsychopharmacology 34(13):2699–2709. 10.1038/npp.2009.101 19675536 10.1038/npp.2009.101 PMC2881681

[CR60] Paulus MP, Stein MB, Simmons AN, Risbrough VB, Halter R, Chaplan SR (2021) The effects of FAAH inhibition on the neural basis of anxiety-related processing in healthy male subjects: a randomized clinical trial. Neuropsychopharmacology 46(5):1011–1019. 10.1038/s41386-020-00936-w 33335310 10.1038/s41386-020-00936-w PMC8105363

[CR61] Perini I, Mayo LM, Capusan AJ, Paul ER, Yngve A, Kampe R, Gauffin E, Mazurka R, Ghafouri B, Stensson N, Asratian A, Hamilton JP, Kastbom Å, Gustafsson PA, Heilig M (2023) Resilience to substance use disorder following childhood maltreatment: association with peripheral biomarkers of endocannabinoid function and neural indices of emotion regulation. Mol Psychiatry. 10.1038/s41380-023-02033-y 37041416 10.1038/s41380-023-02033-y PMC10611562

[CR62] Perkins MN, Stone TW (1982) An iontophoretic investigation of the actions of convulsant kynurenines and their interaction with the endogenous excitant quinolinic acid. Brain Res 247(1):184–187. 10.1016/0006-8993(82)91048-4 6215086 10.1016/0006-8993(82)91048-4

[CR63] Petrie GN, Nastase AS, Aukema RJ, Hill MN (2021) Endocannabinoids, cannabinoids and the regulation of anxiety. Neuropharmacology 195:108626. 10.1016/j.neuropharm.2021.108626 34116110 10.1016/j.neuropharm.2021.108626

[CR64] Rossi S, De Chiara V, Musella A, Kusayanagi H, Mataluni G, Bernardi G, Usiello A, Centonze D (2008) Chronic psychoemotional stress impairs cannabinoid-receptor-mediated control of GABA transmission in the striatum. J Neurosci 28(29):7284–7292. 10.1523/JNEUROSCI.5346-07.2008 18632932 10.1523/JNEUROSCI.5346-07.2008 PMC6670398

[CR65] Russi P, Alesiani M, Lombardi G, Davolio P, Pellicciari R, Moroni F (1992) Nicotinylalanine increases the formation of kynurenic acid in the brain and antagonizes convulsions. J Neurochem 59(6):2076–2080. 10.1111/j.1471-4159.1992.tb10097.x 1431895 10.1111/j.1471-4159.1992.tb10097.x

[CR66] Salter M, Pogson CI (1985) The role of tryptophan 2,3-dioxygenase in the hormonal control of tryptophan metabolism in isolated rat liver cells. Effects of glucocorticoids and experimental diabetes. Biochem J 229(2):499–504. 10.1042/bj2290499 3899109 10.1042/bj2290499 PMC1145083

[CR67] Schreier HMC, Kuras YI, McInnis CM, Thoma MV, St Pierre DG, Hanlin L, Chen X, Wang D, Goldblatt D, Rohleder N (2020) Childhood physical neglect is associated with exaggerated systemic and intracellular inflammatory responses to repeated psychosocial stress in adulthood. Front Psychiatry. 10.3389/fpsyt.2020.00504 32581878 10.3389/fpsyt.2020.00504 PMC7290130

[CR68] Schwandt ML, Heilig M, Hommer DW, George DT, Ramchandani VA (2013) Childhood trauma exposure and alcohol dependence severity in adulthood: mediation by emotional abuse severity and neuroticism. Alcohol Clin Exp Res 37(6):984–992. 10.1111/acer.12053 23278300 10.1111/acer.12053 PMC3620963

[CR69] Schwarcz R, Köhler C (1983) Differential vulnerability of central neurons of the rat to quinolinic acid. Neurosci Lett 38(1):85–90. 10.1016/0304-3940(83)90115-5 6225037 10.1016/0304-3940(83)90115-5

[CR70] Schwarcz R, Whetsell WO, Mangano RM (1983) Quinolinic acid: an endogenous metabolite that produces axon-sparing lesions in rat brain. Sci (New York N Y) 219(4582):316–318. 10.1126/science.6849138 10.1126/science.6849138 6849138

[CR71] Schwarcz R, Bruno JP, Muchowski PJ, Wu H-Q (2012) Kynurenines in the mammalian brain: when physiology meets pathology. Nat Rev Neurosci 13(7):465–477. 10.1038/nrn3257 22678511 10.1038/nrn3257 PMC3681811

[CR72] Secci ME, Auber A, Panlilio LV, Redhi GH, Thorndike EB, Schindler CW, Schwarcz R, Goldberg SR, Justinova Z (2017) Attenuating nicotine reinforcement and relapse by enhancing endogenous brain levels of kynurenic acid in rats and squirrel monkeys. Neuropsychopharmacology 42(8):1619–1629. 10.1038/npp.2017.21 28139681 10.1038/npp.2017.21 PMC5518900

[CR73] Sheehan DV, Lecrubier Y, Sheehan KH, Amorim P, Janavs J, Weiller E, Hergueta T, Baker R, Dunbar GC (1998) The Mini-International Neuropsychiatric Interview (M.I.N.I.): The development and validation of a structured diagnostic psychiatric interview for DSM-IV and ICD-10. J Clin Psychiatry, 59(Suppl 20):22–33;quiz 34–57 9881538

[CR74] Smeets T, Cornelisse S, Quaedflieg CWEM, Meyer T, Jelicic M, Merckelbach H (2012) Introducing the Maastricht acute stress test (MAST): a quick and non-invasive approach to elicit robust autonomic and glucocorticoid stress responses. Psychoneuroendocrinology 37(12):1998–2008. 10.1016/j.psyneuen.2012.04.012 22608857 10.1016/j.psyneuen.2012.04.012

[CR75] Stensson N, Ghafouri B, Gerdle B, Ghafouri N (2017) Alterations of anti-inflammatory lipids in plasma from women with chronic widespread pain—a case control study. Lipids Health Dis 16:112. 10.1186/s12944-017-0505-7 28606089 10.1186/s12944-017-0505-7 PMC5469054

[CR76] Stocchero BA, Rothmann LM, Portolan ET, Lopes TG, Ferraz-Rodrigues C, Garcia MG, de Magalhães Narvaez JC, Grassi-Oliveira R, Viola TW (2024) The consequences of childhood maltreatment on dual-diagnosis psychiatric conditions and clinical outcomes in substance use disorders: a systematic review and meta-analysis. Child Abuse Negl 158:107085. 10.1016/j.chiabu.2024.107085 39418865 10.1016/j.chiabu.2024.107085

[CR77] Stone TW, Perkins MN (1981) Quinolinic acid: a potent endogenous excitant at amino acid receptors in CNS. Eur J Pharmacol 72(4):411–412. 10.1016/0014-2999(81)90587-2 6268428 10.1016/0014-2999(81)90587-2

[CR78] Trepci A, Imbeault S, Wyckelsma VL, Westerblad H, Hermansson S, Andersson DC, Piehl F, Venckunas T, Brazaitis M, Kamandulis S, Brundin L, Erhardt S, Schwieler L (2020) Quantification of plasma kynurenine metabolites following one bout of sprint interval exercise. Int J Tryptophan Res 13:1178646920978241. 10.1177/1178646920978241 33354112 10.1177/1178646920978241 PMC7734489

[CR79] Vamos E, Pardutz A, Klivenyi P, Toldi J, Vecsei L (2009) The role of kynurenines in disorders of the central nervous system: possibilities for neuroprotection. J Neurol Sci 283(1):21–27. 10.1016/j.jns.2009.02.326 19268309 10.1016/j.jns.2009.02.326

[CR80] Vengeliene V, Cannella N, Takahashi T, Spanagel R (2016) Metabolic shift of the kynurenine pathway impairs alcohol and cocaine seeking and relapse. Psychopharmacology 233(18):3449–3459. 10.1007/s00213-016-4384-9 27475106 10.1007/s00213-016-4384-9

[CR81] Wamsteeker JI, Kuzmiski JB, Bains JS (2010) Repeated stress impairs endocannabinoid signaling in the paraventricular nucleus of the hypothalamus. J Neurosci 30(33):11188–11196. 10.1523/JNEUROSCI.1046-10.2010 20720126 10.1523/JNEUROSCI.1046-10.2010 PMC6633493

[CR82] Wang M, Hill MN, Zhang L, Gorzalka BB, Hillard CJ, Alger BE (2012) Acute restraint stress enhances hippocampal endocannabinoid function via glucocorticoid receptor activation. J Psychopharmacol 26(1):56–70. 10.1177/0269881111409606 21890595 10.1177/0269881111409606 PMC3373303

[CR83] Wang Z, Huang S, Li L, Wen Y, Shang D (2023) Kynurenine metabolite changes in individuals with alcohol use disorder: a systematic review and meta-analysis. Drug Alcohol Depend 249:110821. 10.1016/j.drugalcdep.2023.110821 37327508 10.1016/j.drugalcdep.2023.110821

[CR84] Wences Chirino T, Rangel López E, Angulo L, Carrillo Mora A, Landa Solis P, Samudio Cruz C, Bello MAF, Paniagua AC, Pérez R, Ríos Martínez J, Sánchez Chapul L (2023) Crosstalk between Exercise-Derived endocannabinoidome and kynurenines: potential target therapies for obesity and depression symptoms. Pharmaceuticals 16(10). 10.3390/ph16101421 10.3390/ph16101421 PMC10609722 37895892

[CR85] Winters ND, Bedse G, Astafyev AA, Patrick TA, Altemus M, Morgan AJ, Mukerjee S, Johnson KD, Mahajan VR, Uddin MJ, Kingsley PJ, Centanni SW, Siciliano CA, Samuels DC, Marnett LJ, Winder DG, Patel S (2021) Targeting diacylglycerol lipase reduces alcohol consumption in preclinical models. J Clin Invest 131(17):e146861. 10.1172/JCI146861 34292886 10.1172/JCI146861 PMC8409586

[CR86] Yoshida R, Hayaishi O (1978) Induction of pulmonary indoleamine 2,3-dioxygenase by intraperitoneal injection of bacterial lipopolysaccharide. Proc Natl Acad Sci USA 75(8):3998–4000. 10.1073/pnas.75.8.3998 279015 10.1073/pnas.75.8.3998 PMC392917

[CR87] Yoshida R, Urade Y, Tokuda M, Hayaishi O (1979) Induction of indoleamine 2,3-dioxygenase in mouse lung during virus infection. Proc Natl Acad Sci USA 76(8):4084–4086. 10.1073/pnas.76.8.4084 291064 10.1073/pnas.76.8.4084 PMC383982

[CR88] Zhong X, Ming Q, Dong D, Sun X, Cheng C, Xiong G, Li C, Zhang X, Yao S (2019) Childhood maltreatment experience influences neural response to psychosocial stress in adults: an fMRI study. Front Psychol 10:2961. 10.3389/fpsyg.2019.02961 31993010 10.3389/fpsyg.2019.02961 PMC6971063

[CR89] Zhou Q, Sheng M (2013) NMDA receptors in nervous system diseases. Neuropharmacology 74:69–75. 10.1016/j.neuropharm.2013.03.030 23583930 10.1016/j.neuropharm.2013.03.030

