# Supplementary material for: Individual and additive effects of childhood maltreatment and substance use disorder histories on baseline and stress-induced changes in peripheral stress biomarkers
Source: Psychopharmacology (Berl). 2025 Dec 1;243(2):443–56. doi: 10.1007/s00213-025-06953-1 (PMC12904884; doi:10.1007/s00213-025-06953-1)
Supplement: Supplementary file 1 — Supplementary Material 1 (DOCX 25.3 KB) [file 213_2025_6953_MOESM1_ESM.docx]

**TITLE: Individual and additive effects of childhood maltreatment and substance use disorder histories on baseline and stress-induced changes in peripheral stress biomarkers**

**AUTHORS:** Abigail R. Lunge^1^, Lars Östman^2^, Ryann Tansey^1^, Daniel J.O. Roche^3^, Elisabeth R. Paul^2^, Andrea J. Capusan^2^, Markus Heilig^2^, Leah M. Mayo^1^

**Supplementary materials**

Table 3. Summary of ANOVA results

| **Factor** | **CM Status**  **C1** | | **SUD Status**  **Co** | | **CM*SUD**  **C** | |
| --- | --- | --- | --- | --- | --- | --- |
|  | F | p | F | p | F | p |
| Factor 1 | 0.351 | 0.555 | 0.007 | 0.932 | 1.433 | 0.231 |
| Factor 2 | 2.18 | 0.149 | 0.343 | 0.559 | 0.876 | 0.352 |
| Factor 3 | 0.154 | 0.695 | 1.728 | 0.192 | 0.323 | 0.57 |
| Factor 4 | 0.73 | 0.394 | 0.362 | 0.549 | 1.533 | 0.218 |
| Factor 5 | 4.691 | 0.033 | 2.08 | 0.158 | 0.451 | 0.504 |
| Factor 6 | 1.514 | 0.221 | 1.373 | 0.244 | 0 | 0.998 |
| Factor 7 | 2.672 | 0.105 | 0.432 | 0.513 | 1.331 | 0.251 |
| Factor 8 | 1.163 | 0.283 | 0.54 | 0.479 | 3.901 | 0.051 |
| Factor 9 | 0.192 | 0.662 | 5.544 | 0.7 | 4.427 | 0.038 |

Table 3. Summary of ANOVA results for CM and SUD status’ individual and additive effects on factor analysis factors.
